# Tabetic arthropathy revealing neurosyphilis: a new observation

**DOI:** 10.11604/pamj.2014.18.198.4893

**Published:** 2014-07-05

**Authors:** Siham Lakjiri, Fatima Zahra Mernissi

**Affiliations:** 1 Department of Dermatology, CHU Hassan II, Fez, Morocco

**Keywords:** Tabetic arthropathy, neurosyphilis, syphilis, knee

## Image in medicine

A 43-year-old man, consulted for a gradual increase in the volume of the knee that has evolved over 4 years. At the clinical exam the right knee joint was completely pain free with important swelling. The radiological features were characteristic in the right knee joint with complete destruction and slight at the left knee. The diagnosis was confirmed by the presence of positive syphilis serology in the blood, the joint fluid and the cerebrospinal fluid. The patient was treated by penicillin G. Tabès is a rare degenerative manifestation of tertiary syphilis, affecting the posterior column of the spinal cord, occuring on an average of 15 years after contamination. Clinically it is characterized by several manifestations including Charcot arthropathy. The pathophysiology of tabetic arthrpathy is complex. Clinically, there is a significant joint deformation with spontaneous fracture dislocation contrasting with no pain, which delays the consultation and therefore the management (case of our patient who consults after 4 years). The large joints of lower lumbs are its elective location. Orthopedic treatment remains disappointing and management is based on intravenous penicillin G. Value of the prevention, screening and the early treatment of syphilis in order to avoid such a dreaded complication.

**Figure 1:**
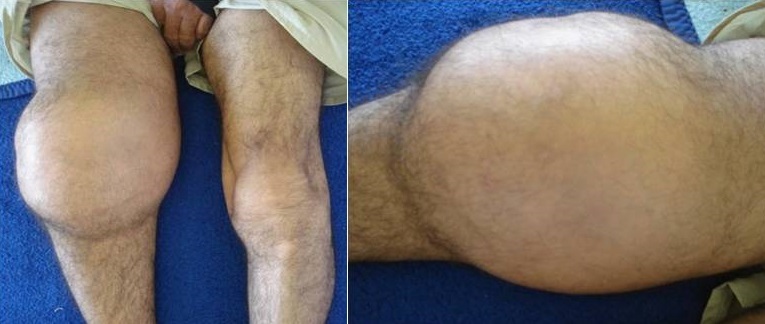
Hydarthrosis of the right knee

